# Initial multi-target approach shows importance of improved caprine arthritis-encephalitis virus control program in Russia for hobbyist goat farms

**DOI:** 10.14202/vetworld.2021.1718-1726

**Published:** 2021-07-01

**Authors:** Eduard A. Shuralev, Nail I. Khammadov, Konstantin A. Osyanin, Inna A. Elizarova, Gaysha R. Salmanova, Nikolai D. Shamaev, Sergei V. Petrov, Clare Whelan, Nikolai Yu. Saushkin, Jeanne V. Samsonova, Ilsur G. Galimzyanov, Marina A. Efimova, Kamil S. Khaertynov, Tagir Kh. Faizov, Malik N. Mukminov, Arkadiy V. Ivanov

**Affiliations:** 1 Department of Applied Ecology, Institute of Environmental Sciences, Kazan Federal University, Kazan, Tatarstan, 420008, Russian Federation; 2 Federal Center for Toxicological, Radiation and Biological Safety, Nauchniy Gorodok-2, Kazan, Tatarstan, 420075, Russian Federation; 3 Central Research Laboratory, Kazan State Medical Academy – Russian Medical Academy of Continuous Professional Education, Kazan, Tatarstan, 420012, Russian Federation; 4 Department of Epizootology and Parasitology, Kazan State Academy of Veterinary Medicine named after N.E. Bauman, Kazan, Tatarstan, 420029, Russian Federation; 5 The United Graduate School of Veterinary Sciences, Gifu University, Yana 1-1, Gifu-city, 501-1193, Japan; 6 Research and Development Department, Enfer Scientific, Naas, Co. Kildare, Ireland; 7 Department of Chemistry, Lomonosov Moscow State University, 1-11 Leninskie Gory, Moscow, 119991, Russian Federation; 8 Department of Surgery, Obstetrics, and Pathology of Companion Animals, Kazan State Academy of Veterinary Medicine named after N.E. Bauman, Kazan, Tatarstan, 420029, Russian Federation; 9 Department of Agricultural Sciences, Russian Academy of Sciences, 32A Leninsky Prospect, Moscow, 119334, Russian Federation

**Keywords:** antibodies, antigens, caprine arthritis-encephalitis virus, goat, proviral DNA

## Abstract

**Background and Aim:**

Several reports described the detection of specific caprine arthritis-encephalitis virus (CAEV) antibodies in Russian goat populations, which indicates the circulation of CAEV in Russian goat farms. The aim of this study was to use a multi-target approach to testing with both serological tests and an in-house real-time (RT) molecular test to investigate the prevalence of CAEV in goats from three hobbyist farms in the Republic of Tatarstan, Russia.

**Materials and Methods:**

We applied a multi-target approach to testing with both enzyme-linked immunosorbent assay (ELISA) and an in-house RT polymerase chain reaction test to investigate the prevalence of CAEV in goats. Animals from the three hobbyist farms were used in this study. The animals from two farms (n=13 for F1 and n=8 for F2) had clinical signs of arthritis and mastitis. In the third farm (n=15 for F3), all goats were home-bred and had no contact with imported animals.

**Results:**

CAEV antibodies (ELISA targets TM *env* and *gag* genes) were detected in serum samples from two farms (F1 and F2), indicating seroprevalence of 87.50-92.31%. Specific CAEV antibodies were also detected in milk samples. CAEV proviral DNA was detected in 53.85-62.50%. The results from all tests performed in the third farm (F3) were negative, indicating that all tests were 100% specific.

**Conclusion:**

The results showed that CAEV is circulating and present in small hobbyist goat farms in Russia. Serological and molecular tests could be important for programs to control and eradicate CAEV in Russia for hobbyist goat farms.

## Introduction

Caprine arthritis-encephalitis virus (CAEV) belongs to the small ruminant lentiviruses (SRLVs), the genus *Lentivirus*, and the family Retroviridae, and can cause serious economic problems for goat farms. The infection may develop into multisystemic inflammatory diseases, which affect the central nervous system in kids and joints and mammary glands in adult goats [1,2]. However, the asymptomatic period may last several months or more. CAEV was initially isolated from an infected adult goat in the United States more than 40 years ago [3]. Since that initial report, the prevalence of CAEV has been reported in many countries [4-10]. Several reports describe the detection of specific CAEV antibodies in Russian goat populations, which indicates the circulation of CAEV in Russian goat farms [11-14]. In Belgium, SRLVs, including CAEV, were detected in small numbers of sheep and goats on hobbyist farms under an ongoing voluntary testing scheme [15], indicating that a low uptake of the voluntary scheme can create difficulties and slow progress in the control program by harboring undetected seropositive animals. Meanwhile, the disproportionately high seroprevalence of CAEV in dwarf goats as reported in Switzerland indicates that these hobby breeds do not fall under official controls [16] and are going undetected; however, these hobby breeders are more likely to inadvertently escape some of the official controls. A widespread of CAEV infection in goat herds in southern Spain has been reported to be associated with factors such as herd size, existence of kidding area, absence of cleaning and disinfection programs, natural mating, and multiparity [5].

A major route for the spread of CAEV infection is colostrum and milk from seropositive goats. In these secretions, free virus and infected macrophages or epithelial cells can be present [17,18]. Cross-species transmission of CAEV has also been observed in wild small ruminants [19].

There is no “gold standard diagnostic test” currently available for CAEV, and the use of a multi-faceted screening approach using both serological and molecular biology techniques for blood and milk samples is recommended to detect positive animals [20,21]. In chapter 3.7.2 of the World Organization for Animal Health (OIE) Terrestrial manual [22], the use of different diagnostic methods, including serology and polymerase chain reaction (PCR), is recommended, along with clinical evaluation and postmortem examinations for diagnosing this persistent infection. Since CAEV is a life-long infection, animals are considered carriers, and present as persistently seropositive.

Antibody responses during CAEV infection do not play a protective role [23], but can be used for diagnostic purposes. In the humoral immune response of goats, immunoglobulin subtype IgG1 is the dominant type in infected goats with clinical arthritis and inflammatory joint lesions [24]. During the SRLV infection process, antibodies against several antigens develop, including capsid protein p25CA, transmembrane protein gp46TM, nucleocapsid p14NC, matrix protein p16MA, and surface protein gp135SU [25]. Due to this antigenic heterogeneity, the use of all or most of these antigens has the potential to increase the sensitivity of CAEV serodiagnosis [26-28].

CAEV, like all members of the retrovirus family, is an RNA-containing pathogen, which, on infection of an organism, integrates a proviral insertion into the genome of the infected animal. Both provirus and virus can be detected by PCR and RT-PCR, respectively [29,30]. CAEV detection methods based on defining the proviral insertion provide the most expedient approach for its detection. CAEV was previously detected using nested PCR in the Philippines [5] and Argentina [9]. For the detection of proviral CAEV DNA, recombinase polymerase amplification and a lateral flow dipstick assay were recommended for use in another study [31]. Detecting CAEV proviral DNA in goat samples could be useful in eradication programs and epidemiological studies.

In Russia, the goat sector is small and consists mostly of hobbyist farmers keeping small numbers of animals on each farm. The current lack of CAEV prevalence data in this hobbyist sector makes it difficult to evaluate the risk of CAEV transmission, even for such relatively low numbers of farms and animals. Here, we report the prevalence of CAEV in goats from three hobbyist farms as determined by enzyme-linked immunosorbent assay (ELISA) and real-time (RT) PCR in the Republic of Tatarstan, Russia.

## Materials and Methods

### Ethical approval

Bioethics Committee of Federal Center for Toxicological, Radiation and Biological Safety provided full approval for this research. Special ethical approval was not required because animals were not involved in an experimental study. Only blood and milk samples, collected by veterinarians, were used. The samples taken did not exceed the volume that would have been taken for routine veterinary/animal husbandry purposes.

### Study period and location

The study was conducted at three hobbyist goat farms in the Republic of Tatarstan, Russia, in 2015, during routine veterinary examination of farms.

### Animals and clinical samples

**Figure-1:**
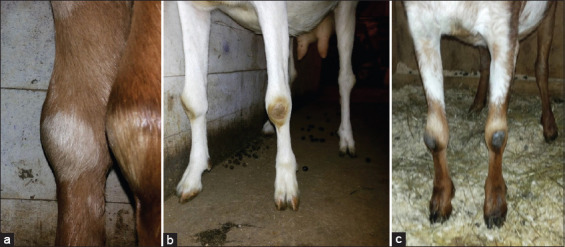
Clinical signs of arthritis in goats from farms F1 (a, b) and F2 (c).

Animals from two farms (n=13 for F1, n=8 for F2) containing a mixture of home-bred and purchased animals, some of which were already showing clinical signs of arthritis (Figure-1) and mastitis, were used here to assess the prevalence of infection. Goats from a third farm (n=15 for F3) were used to determine the tests’ apparent specificity, which were all home-bred and had no contact with imported animals. They showed no clinical signs of CAEV. No CAEV testing history was available for the animals used in this study.

Whole blood was collected into 4.5 mLVacuette^®^ K3E K3EDTA 13×75 lavender cap-black ring, premium tubes(Greiner Bio-One GmbH, Austria), and also into Vacuette^®^ Tube 4.5 mL Z Serum Clot Activator 13×75 red cap-black ring, premium tubes (Greiner Bio-One GmbH, Austria) from the jugular vein using Vacuette^®^ Multiple Use Drawing Needles, 18G×1 1/2” pink, sterile, latex-free, 1.25×38 mm (Greiner Bio-One GmbH, Austria). The serum was separated in the Serum Clot activator tubes by centrifugation at 500 g for 15 min. Whole blood and serum samples were stored at −20°C.

Milk samples were collected in glass tubes (Khimlaborpribor, Russia) and then stored at 2–8°C for 24 h. Samples were then centrifuged at 500 g for 15 min, defatted, and stored at −20°C.

### Antibody detection

Goat serum and milk samples were tested using the commercial ELISA Maedi-Visna/CAEV Antibody Test Kit (IDEXX, France) with one modification for milk testing. This ELISA uses a mixture of a synthetic peptide of the immunogenic region of the transmembrane protein (TM *env* gene) and recombinant p28 protein, which is part of the viral capsid (*gag* gene), immobilized as an antigen in the wells of an ELISA plate. Briefly, serum samples were diluted 1:20 and individual milk samples were diluted 1:50 in dilution buffer and mixed before following the manufacturer’s instructions for the ELISA test for serum samples. Results were analyzed in accordance with the manufacturer’s instructions and are presented as S/P %.

### Extraction of nucleic acids

For the extraction of nucleic acids, 1 mL of milk sample was placed into a centrifuge tube and centrifuged at 7000 rpm (MiniSpin, Eppendorf, Germany) for 5 min, 800 μL of supernatant was removed, and the remaining 200 μL was used for DNA extraction. Whole blood samples were used without centrifugation.

The AmpliPraym DNA-sorb-B kit (NextBio, USA) was used in accordance with the manufacturer’s instructions for the isolation of DNA from milk and whole blood samples. Lysis solution (300 μL) and 100 μL of milk or whole blood sample were used per extraction.

The extracted DNA concentration and purity were measured using a UV5Nano spectrophotometer (Mettler Toledo, Switzerland), in accordance with the manufacturer’s instructions. Samples of nucleic acids were stored at −80°C until use.

### Design of oligonucleotide primers

**Figure-2:**
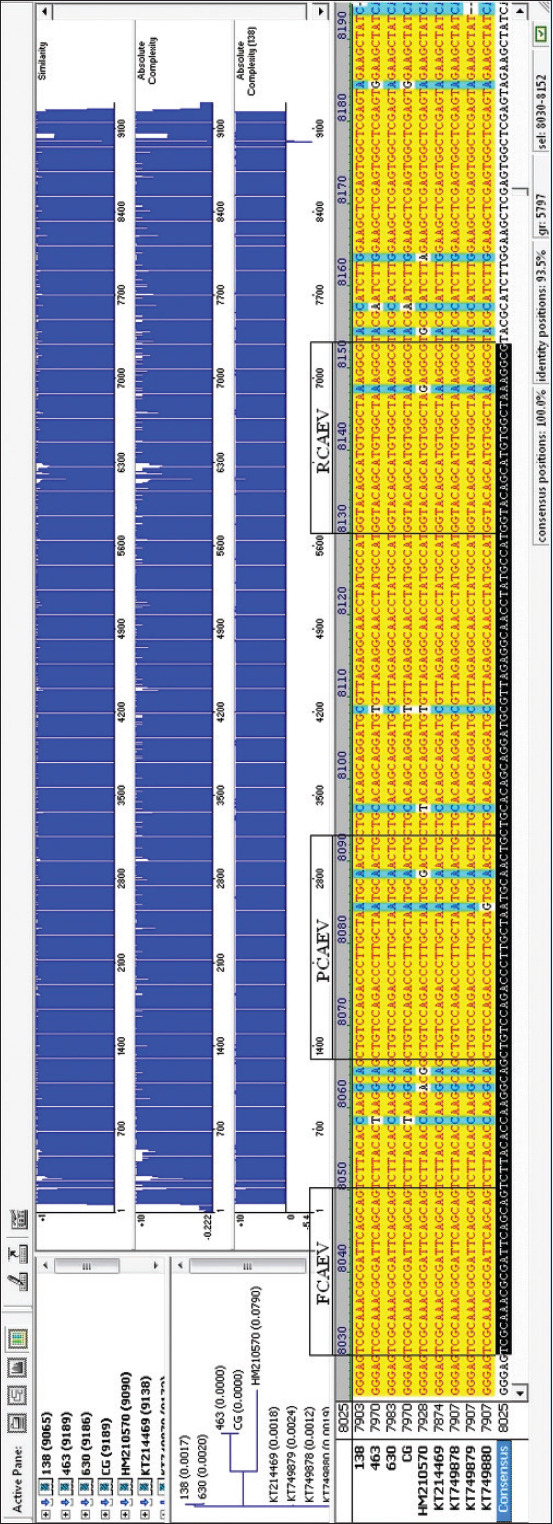
Homologous portion of the proviral DNA or viral RNA in caprine arthritis–encephalitis virus (CAEV) isolates/strains with positions in CAEV genome shown.

For the in-house RT-PCR assay, the target site from CAEV proviral DNA, available in GenBank (GenBank accession number: NC_001463), was selected using AlignX (ClustalW, Conway Institute, UCD, Ireland) and Vector NTI Version 9.1 (Invitrogen, USA) programs. Several isolates/strains of the virus were analyzed to identify a portion of the DNA that was homologous across all (Figure-2). The *env* gene was selected, Standard Nucleotide BLAST (https://blast.ncbi.nlm.nih.gov) was used and identified a region between 7975 and 8098 bp (GenBank accession number: NC_001463) as highly specific, and primers and probes were designed within this region.

The primer set (forward FCAEV; reverse RCAEV) and probe (PCAEV) used in this study (Table-1) were designed based on the CAEV proviral DNA specific region identified as described above. Using Standard Nucleotide BLAST, it was confirmed that, in this format, no cross-reaction with DNA of other organisms can be observed and it is 100% specific to CAEV. The Oligo Analysis module of Vector NTI was used to check the primers *in vivo* before syntheses (e.g. melting temperature [Tm], primer-dimer formation, and primer self-complementarity). The maximum annealing temperature of the primers was 60.3°C for forward (F) and 60.3°C for reverse (R) primers. The annealing temperature of the probe (P) was 65.5°C. The designed primers were found not to form dimers, secondary structures, or palindromes and had GC composition of 40-60%. The primers and probe (labeled with reporter and quencher dye [ROX, BHQ2] at its 5′ and 3′ ends, respectively) were synthesized by Syntol (Moscow, Russia) (Table-1).

**Table-1:** Primers and probe designed in this study.

Primers and probe	Sequence (5’-3’)	Position in genome^1^	Amplicon size (bp)
FCAEV	TCGCAAACGCGATTCAGCAGT	7975-7995	124
PCAEV	ROX-CTGTCCAGACCCTTGCTAATGCAACTGC-BHQ2	8011-8038	
RCAEV	ACGCCTTTAGCCACATGCTGTACC	8075-8098	

1 Numbering according to the Caprine arthritis-encephalitis virus, complete genome (GenBank accession number: NC_001463). CAEV=Caprine arthritis-encephalitis virus

### RT-PCR

RT-PCR for CAEV proviral DNA was performed using a universal master mix RT-PCR kit (Syntol, Moscow, Russia), comprising: 25 mM MgCl_2_, 2.5 mM dNTP, PCR buffer ×10, Taq polymerase, and deionized water. The final volume of 20 μL of the PCR mixture contained: 1.5 μL of 25 mM MgCl_2_ solution, 0.5 μL of 10 pM probe solution, 0.5 μL of 10 pM of each primer solution, 1.5 μL of 2.5 mM dNTP solution, 1.5 μL of 10× buffer for PCR, 0.5 μL of Taq polymerase, 10 μL of DNA extract, and 3.5 μL of deionized water. PCR was carried out in RT on the amplification platform C1000 with an optical reaction module, CFX96 (Bio-Rad). The PCR cycling conditions were as follows: (I) Denaturation at 95°C for 3 min, followed by (II) 5 cycles of 10 s each at 95°C and 30 s at 60.0°C, and then (III) 39 cycles of 10 s at 95°C and 30 s at 60.0°C (acquisition of fluorescent signal).

### Positive control

A synthetic insert of 150 bp of synthetic DNA(5′-gcaagtctgggagtcgcaaacgcgattcagcagtcctatactagggcggctgtccagacccttgctaatgcaactgctgcacagcaggatgtgttagaagcaacctatgccatggtacagcatgtggctaaaggcgtcaggatcttggaa-3′) was designed to include recognition sites for the CAEV gene (GenBank accession number: NC_001463) and was inserted into synthetic oligonucleotide sequences. The final nucleotide sequence was synthesized and then subcloned within plasmid pAL2-T (ZAO Evrogen, Russia), which was used as a positive control in RT-PCR for CAEV proviral DNA.

### Statistical analysis

The prevalence was calculated using Wilson 95% confidence interval (CI) without correction for continuity available online (The CI of a Proportion/VassarStats: http://vassarstats.net/prop1.html). The agreement between tests was calculated using Kappa available online (Kappa as a Measure of Concordance in Categorical Sorting/VassarStats: http://vassarstats.net/kappa.html).

## Results

### Seroprevalence of CAEV

For comparison of the CAEV seroprevalence in the two herds where animals were exhibiting clinical symptoms of CAEV infection (Figure-1), the results from F1 and F2 were analyzed. In the cases of F1 and F2, 12/13 and 7/8 goats were positive in the IDEXX assay, with seroprevalence of 92.31% (95% CI 66.69-98.63%) and 87.50% (95% CI 52.91-97.76%), respectively. For comparison of the apparent specificity, 15 goats from F3 were tested for the presence of specific CAEV antibodies in serum samples using the Maedi-Visna/CAEV Antibody Test Kit (IDEXX). None of these 15 goats was antibody-positive by ELISA (Table-2).

**Table-2:** Summary of all test results using the IDEXX Maedi-Visna/CAEV Antibody Test Kit and in-house RT-PCR assay.

Farm	Animal ID	CAEV Symptoms	IDEXX ELISA, Serum	PCR, Blood	IDEXX ELISA, Milk	PCR, Milk
Farm 1 (F1)	F1-1	Present	POS	POS	NEG	POS
	F1-2	Present	POS	NEG	POS	NEG
	F1-3	Present	POS	NEG	POS	NEG
	F1-4	Present	POS	POS	POS	NEG
	F1-5	Present	POS	NEG	POS	NEG
	F1-6	Absent	POS	POS	POS	POS
	F1-7	Absent	POS	POS	POS	POS
	F1-8	Present	POS	NEG	N/A	N/A
	F1-9	Present	POS	NEG	POS	NEG
	F1-10	Present	POS	POS	N/A	N/A
	F1-11	Present	POS	POS	POS	POS
	F1-12	Present	POS	POS	POS	NEG
	F1-13	Absent	NEG	NEG	N/A	N/A
Far 2 (F2)	F2-1	Present	POS	POS	POS	POS
	F2-2	Present	POS	POS	N/A	N/A
	F2-3	Absent	POS	POS	N/A	N/A
	F2-4	Present	POS	POS	N/A	N/A
	F2-5	Present	POS	NEG	N/A	N/A
	F2-6	Absent	POS	POS	N/A	N/A
	F2-7	Absent	NEG	NEG	NEG	NEG
	F2-8	Absent	POS	NEG	NEG	NEG
Farm 3 (F3)	F3-1	Absent	NEG	NEG	N/A	N/A
	F3-2	Absent	NEG	NEG	N/A	N/A
	F3-3	Absent	NEG	NEG	NEG	NEG
	F3-4	Absent	NEG	NEG	N/A	N/A
	F3-5	Absent	NEG	NEG	NEG	NEG
	F3-6	Absent	NEG	NEG	NEG	NEG
	F3-7	Absent	NEG	NEG	NEG	NEG
	F3-8	Absent	NEG	NEG	N/A	N/A
	F3-9	Absent	NEG	NEG	N/A	N/A
	F3-10	Absent	NEG	NEG	N/A	N/A
	F3-11	Absent	NEG	NEG	NEG	NEG
	F3-12	Absent	NEG	NEG	N/A	N/A
	F3-13	Absent	NEG	NEG	N/A	N/A
	F3-14	Absent	NEG	NEG	N/A	N/A
	F3-15	Absent	NEG	NEG	N/A	N/A

POS=Positive result, NEG=Negative result, N/A=Not applicable. CAEV=Caprine arthritis-encephalitis virus, ELISA=Enzyme-linked immunosorbent assay, RT-PCR=Real-time Polymerase chain reaction

### Prevalence of CAEV antibody in milk

Positive ELISA results were obtained for 9/10 milk samples from goats of F1 (90.00%, 95% CI 59.59-98.21%). For 2/10 animals, there were no clinical signs, but they were determined to be serum positive. Antibodies were detected in one sample obtained from a goat with clinical symptoms (33.33%; 95% CI 6.15-79.23%). Only 5/15 milk samples from F3 were tested by ELISA, all of which were negative.

### Comparison of serum and milk

The observed agreement between serum and milk test results for IDEXX for the three farms (n=18) was 0.7692, indicating good agreement. However, serum ELISA testing detected more positive goats than the milk samples tested here.

### Synthetic viral DNA detection limit

**Figure-3:**
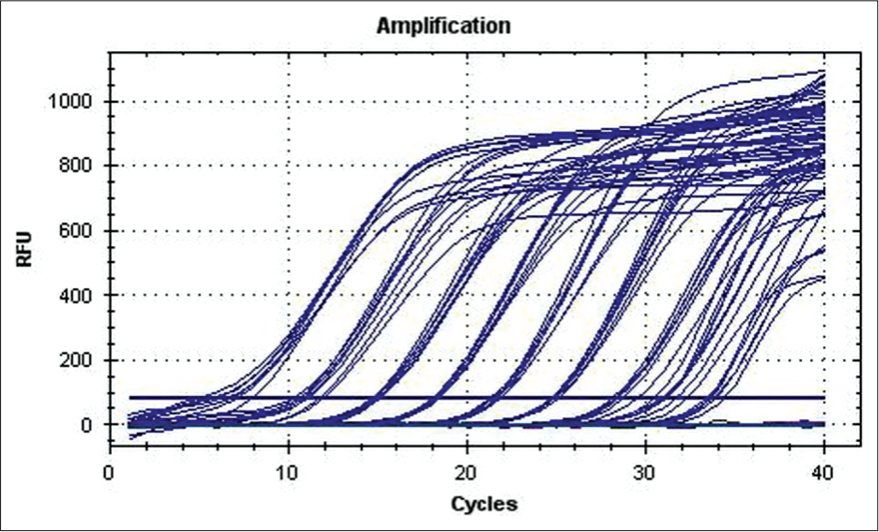
Real-time polymerase chain reaction result of plasmid DNA containing marker DNA caprine arthritis–encephalitis virus (CAEV). Amplification curves of CAEV, respectively, using serial ten-fold dilutions of the mixed recombinant plasmids from, 1×10^11^, 1×10^10^, 1×10^9^, 1×10^8^, 1×10^7^, 1×10^6^, 1×10^5^, 1×10^4^, 1×10^3^, and 100 copies/mL.

To determine the detection limit of the PCR, synthetic viral DNA was titrated (13 ten-fold dilutions) and tested. The initial concentration of the DNA was 426.6 ng/μL (plasmid DNA 3150 bp; 1×10^14^ copies/mL). RT-PCR was carried out on the dilutions, starting at 1×10^11^ copies/mL. Each dilution was amplified and the results were confirmed for eight repeats per dilution (Figure-3).

The last dilution successfully amplified was 1×10^3^ copies/mL (1×10 copies/10 μL); at this dilution, a positive reaction was observed in six out of eight of the repeats, while all repeats of the dilution 1×10^4^ copies/mL (1×10^2^ copies/10 μL) were amplified in all cases. The dilution 100 copies/mL (1×1 copies/10 μL) did not amplify. To evaluate the inter-assay variability, ten-fold dilutions of 1×10^12^, 1×10^11^, 1×10^10^, 1×10^9^, 1×10^8^, 1×10^7^, 1×10^6^, 1×10^5^, 1×10^4^, 1×10^3^, and 100 copies/mL of the plasmid DNA were tested (eight replicates on the same amplification run). The CV values of intra-assay were 0.1-14.88 (Table-3).

**Table-3:** Intra-assay for PCR detection of synthesized viral DNA.

Target	Conc. (copies/reaction)	Number of determinations	Mean Ct	CV
CAEV	1×10^9^	8	6.38	0.827677
	1×10^8^	8	10.98	0.535454
	1×10^7^	8	14.86	0.157619
	1×10^6^	8	18.21	0.103976
	1×10^5^	8	21.57	0.182821
	1×10^4^	8	24.84	0.1283
	1×10^3^	8	28.49	0.414638
	1×10^2^	8	31.19	0.515722
	1×10	8	33.75	14.61552
	1×1	8	N/A	N/A

Mean Ct - average value of the beginning of registration of amplification reaction, CV - coefficient of variation, standard deviation from Mean Ct. CAEV=Caprine arthritis-encephalitis virus, PCR=Polymerase chain reaction

### RT-PCR for CAEV proviral DNA

The total DNA concentrations for both milk and whole blood-based extractions were in the range of 200-1.200 μg/mL, and all DNA extracted in this study had a 260/280 ratio of >1.8 (data not shown).

**Figure-4:**
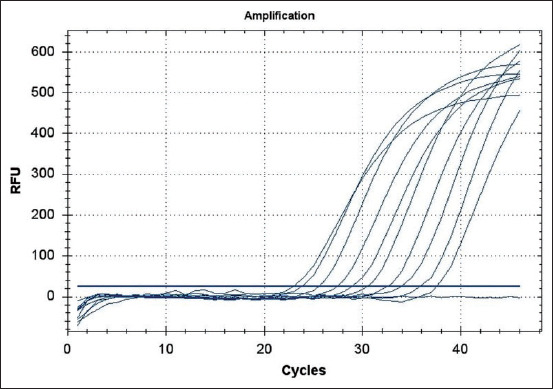
Real-time polymerase chain reaction result of goat F1-11 milk sample: Total DNA titration from 900 to 0.05 μg/mL (900 μg/mL, 300 μg/mL, 100 μg/mL, 33 μg/mL, 11 μg/mL, 3.7 μg/mL, 1.2 μg/mL, 0.41 μg/mL, and 0.05 μg/mL) and no amplification negative control.

To determine the detection limit for the PCR assay, serial dilutions were prepared based on total DNA extracted from a whole blood sample. Serial dilutions of total goat DNA from 900-0.05 μg/mL (Figure-4) were used. The Cq at the lowest DNA dilution (0.05 μg/mL total DNA) was determined as 37.57. Whole blood samples from all 15 goats from F3 were tested and five were tested by the milk-based PCR. In both cases, all samples were PCR-negative (Table-2).

A total of 13 blood samples from F1 were tested by RT-PCR and CAEV proviral DNA was detected in 7/13 animals (Table-2), with a prevalence rate of 53.85% (95% CI 29.15-76.80%). Overall, five of these seven goats had clinical signs of CAEV infection (arthritis); there were no clinical signs in the other two animals. RT-PCR was carried out for ten milk samples from these 13 animals. CAEV proviral DNA was detected in 4/10 milk samples. These four animals were also positive when blood was tested by PCR.

In F2, blood samples from eight goats were tested and PCR was positive in 5/8 samples, indicating the prevalence of CAEV proviral DNA to be 62.50% (95% CI 30.57-86.32%). Of the five animals tested as positive, 3/5 had clinical CAEV symptoms. Milk samples from F2 were also tested for three animals, using RT-PCR. CAEV proviral DNA was detected in 1/3 of these milk samples. This one positive animal was also positive when blood was tested by PCR. CAEV proviral DNA was not detected in any blood or milk samples from F3, where clinical signs of CAEV were absent in all animals. These results indicate that RT-PCR as used in this study, is highly sensitive and can detect CAEV proviral DNA at very low concentrations (Figure-3).

### Comparison of ELISA and PCR

An overall good level of agreement of 0.6182 was determined for samples (milk/serum/blood) from all three farms (n=36) between all tests performed on the IDEXX ELISA and the PCR.

## Discussion

There are no official reports of CAEV infection in goats on hobbyist farms and are currently no eradication/monitoring programs in the Republic of Tatarstan, Russia. As of 2016, there was a goat/sheep population of 65,800 heads [32], of which it is estimated that 90.5% are in small and hobbyist farms. At present, control measures consist only of veterinary certification of imported goats as originating from a CAEV-free region [33], which aims to prevent the introduction of infection into farms in Russia from abroad. However, it is common practice for large dairy goat farmers to strictly control and test for a range of different infections to help prevent entry of any infected animals into their herds. Goats from three small hobbyist farms in the Republic of Tatarstan, Russia, were investigated here for the presence of CAEV antibodies and proviral DNA. CAEV antibodies were detected in serum samples from two farms, where animals were also showing clinical signs of the disease, with seroprevalence at levels of 92.31% (95% CI 66.69-98.63%) in Farm 1 and 87.50% (95% CI 52.91-97.76%) in Farm 2. Specific CAEV antibodies were also detected in milk samples from the two farms (F1 and F2), indicating prevalence rates of 90.00% (95% CI 59.59-98.21%) and 33.33% (95% CI 6.15-79.23%), respectively. CAEV proviral DNA was also detected at rates of 53.85% (95% CI 29.15-76.80%) and 62.50% (95% CI 30.57-86.32%) for the two farms, respectively. The observed agreement between serum and milk ELISA results was 0.7692 and that between ELISA and PCR was 0.6182 in the two farms. This indicates the presence of previously unreported CAEV seropositive goats in these farms in the Republic of Tatarstan, Russia.

An Italian study demonstrated that ELISA can be used as a diagnostic test for control measures, for aiding the reduction of seroprevalence as well as clinical manifestations of CAEV infection [34]. The reactivity of CAEV antigens in serological tests may vary depending on the geographical and breeding origin of the goats [35]. The presence of antibodies and proviral DNA in goat samples may vary with time, and the combination of different tests for CAEV diagnostics may improve the efficacy of control and eradication programs [20,21,36]. Therefore, for epidemiological purposes, serological assays with various CAEV antigens as well as PCR methods are needed for detection of the disease. The study in Thailand showed that the combination of ELISA and PCR provided advantages to detect CAEV-infected goats [37]. The IDEXX ELISA kit, which was used in our study, uses a mixture of the transmembrane protein (TM ENV gene) and recombinant p28 protein (GaG gene) for the detection of the infection as a multi-antigen approach. It was reported that monoclonal antibodies against p28 are also reactive against p55 (gag) protein and the intermediate cleavage products, p44, p36, and p22 [38]. The antibody response was significantly higher among arthritic than asymptomatic goats [28]. In our study, all animals with clinical signs of CAEV were seropositive.

There is potential for using PCR for CAEV, as an alternative to serology or as a supplemental test. CAEV RNA PCR and previously PCR for detecting CAEV proviral DNA were shown to be highly efficient [39]. It was also reported that the prevalence of CAEV proviral DNA and CAEV in the seminal plasma was significantly higher in bucks with PCR-positive blood [40]. Proviral DNA was detectable 15 days after experimental CAEV infection, whereas specific antibodies were detected after 40-60 days using RT-PCR also targeting a specific region of the CAEV *env* gene [41]. The CAEV genome is characterized by pronounced polymorphism of the nucleotide sequence. In the conservative sequence that we chose [42] to indicate the CAEV provirus, polymorphism is also observed (Figure-2), and the oligonucleotides for indicating CAEV are complementary to non-polymorphic regions of this sequence.

The results from this study show that the use of both ELISA and PCR has the advantage of potentially improving the sensitivity. However, the lack of agreement between ELISA and PCR results reported by other authors [20,21] was also observed in this study. The results of this study revealed a pattern of a positive reaction in ELISA and PCR (with both blood samples and milk) in goats with clinical manifestations of the disease (samples: F1-6, F1-7, F1-11, and F2-1). This is associated with the peculiarities of the CAEV infection process. The provirus can be detected at early stages and during virus release into the environment, and antibodies are synthesized at a later stage after infection and can circulate in infected animals for a long time. Recently, it was reported that goats on multispecies farms (where goats and sheep coexist) in Italy had higher CAEV seroprevalence, where sheep can serve as a reservoir of small ruminant lentivirus infection [43]. In addition, indications for cross-species transmission of small ruminant lentivirus strains between sheep and goats were found in Belgium [44]. SRLVs are known to cross-species barriers and infections of goats by sheep and vice versa were previously described [45]. A study in southern France looking at *pol* gene sequences indicated significant genomic heterogeneity between viral variants from goat and sheep isolates, which may lead to them crossing the species barrier [46]. In most cases, hobbyist farms are multispecies farms, and small ruminant lentivirus control programs should be concentrated on both sheep and goats. The lack of regular screening for SRLVs is increasing their spread [47-49].

The results of our study on three small farms in the Republic of Tatarstan indicate the possibility that CAEV is spreading among goats on hobbyist farms, the owners of which lack the toolsto control this viral disease. Moreover, the absence of reported CAEV cases in large goat farms indicates compliance with veterinary and animal husbandry regulations. The initial work presented here on in-house PCR shows that the test is specific in the samples tested; however, further work is needed to develop the assay further for both milk- and blood-based assays. Work analyzing the prevalence of CAEV in other parts of the Russian Federation will continue.

## Conclusion

The results of this study indicate that CAEV is present and circulating in small hobbyist goat farms in the Republic of Tatarstan, Russia, and is currently going undetected in the absence of a control program or monitoring. Due to the complex nature of CAEV infection and the viral life cycle and based on the results of this comparative study, it can be concluded that serological tests, targeting different proteins, as well as molecular-based tests could be important for any future CAEV control and eradication programs in Russia for hobbyist goat farms.

## Authors’ Contributions

EAS, NIK, and AVI: Conceptualization. EAS, NIK, CW, JVS, KSK, and TKF: Data curation. KAO, GRS, NDS, SVP, NYS, IGG, and MAE: Formal analysis. EAS and MNM: Funding acquisition. EAS, NIK, NDS, CW, NYS, JVS, and MAE: Investigation. EAS, NIK, KAO, IAE, GRS, SVP, NYS, and JVS: Methodology. EAS, MNM, and AVI: Project administration. EAS, NIK, NDS, JVS, IGG, and TKF: Resources. NIK: Software. MNM and AVI: Supervision. EAS, NIK, CW, NYS, JVS, and KSK: Validation. NIK, KAO, IAE, NDS, SVP, and IGG: Visualization. EAS, NIK, NDS, CW, JVS, and MAE: Writing – original draft. EAS, NIK, CW, TKF, and MNM: Writing – review and editing. All authors read and approved the final manuscript.
